# Effects of Overlap Length on Flammability and Fire Hazard of Vertical Polymethyl Methacrylate (PMMA) Plate Array

**DOI:** 10.3390/polym12122826

**Published:** 2020-11-27

**Authors:** Weiguang An, Kaiyang Hu, Tao Wang, Lujun Peng, Song Li, Xiangming Hu

**Affiliations:** 1 Jiangsu Key Laboratory of Fire Safety in Urban Underground Space, China University of Mining and Technology, Xuzhou 221116, Jiangsu, China; TS19120073P31@cumt.edu.cn (K.H.); TS19120031A31@cumt.edu.cn (T.W.); TS19120027A31@cumt.edu.cn (L.P.); TS19120014A31TM@cumt.edu.cn (S.L.); 2 State Key Laboratory of Coal Resources and Safe Mining, China University of Mining and Technology, No. 1 University Road, Xuzhou 221116, Jiangsu, China; 3 Key Laboratory of Mining Disaster Prevention and Control, Qingdao 266590, China

**Keywords:** flammability, polymethyl methacrylate, fire hazard, flame dimension, discrete flame spread, temperature field

## Abstract

Polymethyl methacrylate (PMMA) plates are widely used in buildings or factories for natural lighting. Commonly PMMA plates are installed as a discrete array. However, PMMA plates are very susceptible to fire. Therefore, experimental study on flammability and fire hazard of vertical PMMA plate array with different overlap length (*D*) was conducted in this work. The average flame height ($H_{f}$) increases first and then decreases with an increase in the overlap length, and reaches the maximum when *D* = 40 mm. The discrete flame spread speed ($V_{f}$) also rises first and then drops with the increase of *D*, which is mainly due to the heat transfer from the PMMA flame to the next plate. A model for predicting the flame spread rate of discrete PMMA array is established. The predicted results are consistent with experimental ones, with a predicted error smaller than 15%. The average temperature of flame zone rises first and then drops as *D* increases, reaching the maximum when *D* = 40 mm. This leads to the same changing trend of radiative heat flux. Results obtained in this work provide a reference for fire hazard evaluation and fire safety design of PMMA plates employed in buildings or industrial sites.

## 1. Introduction

In recent years, polymethyl methacrylate (PMMA, also referred to as organic glass) has been widely used in industry and buildings due to its advantages, such as better transparency, chemical stability, mechanical properties, weather resistance, easy dyeing, and processing property, as well as graceful appearance. A great number of papers focus on the mechanical and environmental properties of PMMA [1,2]. However, there are relatively fewer studies concerning flammability and fire hazard, especially flame spread characteristics. In fact, PMMA is flammable and emits flammable, explosive, irritating gases during combustion [3], thereby contributing to the rapid flame spread. Thus, it is of great practical significance to conduct experimental study on flammability and fire hazard of the PMMA plate. Results of this work are beneficial to the fire hazard evaluation and fire safety design of industrial sites or buildings employing PMMA.

Most of previous works focused on combustion and flame spread over continuous materials, either solid materials or liquid materials [4,5,6,7,8,9,10,11,12]. Moreover, scholars have conducted extensive research on the flame spread and combustion characteristics of continuous PMMA plate. Zhu et al. [5] experimentally studied the effect of spacing between the wall and the PMMA plate (1 mm thick) on the upward flame spread behavior. Their experiment results indicated that, as the spacing increases, the flame height, pyrolysis height, and burning-out length exhibit the same rule, that is, they first increase and then decrease. Li et al. [6] also studied the effect of the spacing (*L*) between the wall and the PMMA plate on its upward flame spread, and proposed the fitting formulas for the front and back flame height with different spacings. It’s found that when *L* is less than 13 mm, the flame height and flame spread rate are positively correlated with the spacing while negatively correlated when *L* is greater than 13 mm. Previous studies demonstrated that the material size and inclined angle have a significant influence on the flame spread characteristics over the PMMA. Studies from Pizzo et al. [7] shows when the width increases, the flame spread rate over PMMA plate increases accordingly. Tsai [8] experimentally studied the upward flame spread over PMMA sample which is 100, 200, 300, 500, and 700 mm in width, respectively. Studies show that the heat release rate, flame height, and flame temperature per unit width increase as the sample width increases. Dupuy et al. [9] discussed the effect of radiation and convective heat transfer on the flame spread over the PMMA with different inclination angles. Gollner et al. [10] studied the PMMA flame spread rate and the mass loss rate at different inclined angles, and found that the maximum flame spread rate occurred in the vertical case when the mass loss rate at this time was not the maximum. Gong et al. [11] experimentally and theoretically analyzed the heat and mass transfer process of the downward flame spread over PMMA plate with limited width. The study indicates that under two-dimensional conditions, the inverted “V” shape of the flame front accelerates the mass loss rate and flame spread rate of the specimen because the lateral combustion of the specimen and the oxygen diffusion are enhanced. Moreover, the mass loss rate and flame spread rate are functions of temperature, leading edge angle, and specimen size. Consalvi et al. [12] established a mathematical model for upward flame spread over the PMMA, analyzed the combustion and radiation problems in the gas phase in combination with the two-dimensional average turbulence N-S equation, and the relation of pyrolysis trajectory and the heat release rate per unit width to the pyrolysis height was given.

For combustion and flame spread behaviors of discrete combustibles, previous studies are relatively less. An experimental and theoretical study was conducted by Jiang et al. to investigate the controlling mechanisms of flame spread using arrays of wooden dowels, with dowel spacings of 0.75, 0.875, and 1.5 cm [13]. They developed models to predict horizontal flame spread rate and the number of burning wooden dowels. The vertical flame spread behavior of discrete PMMA was studied by Miller and Gollner [14]. They found that the flame spread rate first increased and then decreased with the increase of PMMA coverage, and reached the maximum as the coverage was 0.67.

To sum up, previous studies focused on flammability and flame spread over continuous PMMA plate, while there are few researches on the flammability and fire hazard of discrete PMMA plate. However, PMMA plates are commonly installed or placed as a certain array, which are discrete. Thus, this paper studies flammability and fire hazard of vertical PMMA plate array, analyzes the variation of flame spread rate, flame geometry, heat transfer, and temperature distribution, and reveals the influence mechanism of plate overlap length.

## 2. Experimental Materials and Methods

**Figure 1:**
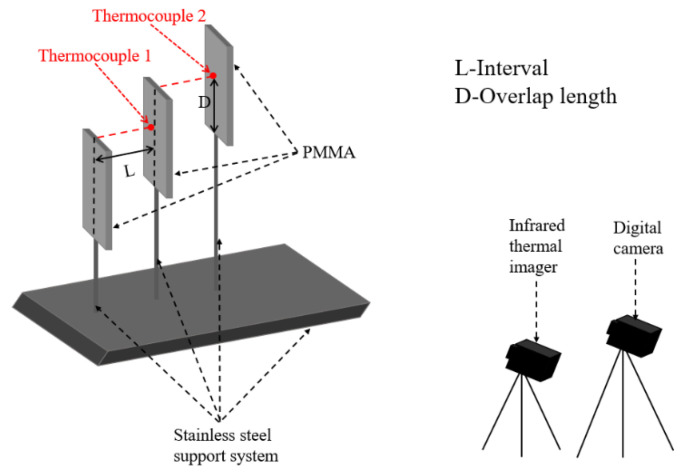
Experimental system.

As shown in Figure 1, the experimental system included K-type thermocouples, data acquisition instruments, cameras, iron frame stages, specimen holders, rotation racks, cross clips, infrared cameras, and computers, etc. The experimental environmental conditions were shown in Table 1. PMMA can be divided into several categories, including casting, injection molding, extrusion, hot molding, etc. The casting PMMA has good thermophysical properties and high transparency, which were widely used in previous works. Therefore, rectangular transparent casting PMMA plates with a length of 100 mm, a width of 40 mm, and a thickness of 5 mm were used in this paper. The PMMA samples were produced by Nanchang Inter Industrial Co. Ltd. (Nanchang, China). No flame retardant was added to the PMMA specimen, and its detailed characteristics were shown in Table 2 [15]. The specific position on the surface of the specimen was marked with dots and parallel lines to facilitate fixing of the K-type thermocouple and adjustment of the position of the temperature measurement point. A non-combustible double-sided adhesive with a length of 20 mm was stuck along the edge on one side of the specimen to prevent the specimen falling off from the rack due to its softening caused by long burning time. The distribution of PMMA plates was shown in Figure 1. The PMMA plates were parallel with a certain spacing, and their vertical centerlines were in the same plane. Moreover, they were distributed in the stepladder mode with a certain overlap length.

The overlapping length of two adjacent plates was changed in the experiment, ensuring that the spacing between the two plates was constant. The method for determining the spacing is explained as follows. Firstly, according to “Code for construction of decoration of housings” (Chinese standard, GB 5024-2002) and “Code for fire prevention in design of interior decoration of buildings” (Chinese standard, GB 50222-2017), the value range of the spacing between two adjacent PMMA plates is determined, i.e., 8–12 mm. Secondly, the preliminary experiment is conducted with fewer samples before the formal experiment to find out the best experimental condition concerning the spacing. It is found that both the flame spread rate and average flame height drop as the spacing rises. Therefore, it is preliminarily deduced that the fire hazard is the most significant when the spacing (*L*) is 8 mm. This fire scene which is the most dangerous is selected for further studying and formal tests. The change range of overlap length (*D*) was set as 20–50 mm. The experimental conditions were listed in the Table 3.

A digital camera with a data acquisition frequency of 25 frame/s and an infrared camera with a data acquisition frequency of 100 frame/s were employed to record the flame spread behavior, including flame height and flame spread rate. The infrared camera and the IR software were produced by Juge Electronics Co. Ltd. (Shanghai, China). The digital camera was produced by SONY (Tokyo, Japan). The performance parameters of these cameras are shown in Table 4 [16]. In order to make the infrared camera data more accurate and reliable, it is necessary to calibrate the emissivity for the measuring software of the infrared camera prior to use. The flame emissivity is affected by many factors, such as material category, environment, smoke concentration. The method to calibrate the emissivity is explained as follows. The temperature distribution of PMMA fire was simultaneously measured by the K-type thermocouple and the infrared camera. The emissivity in the measuring software of the infrared camera must be adjusted to obtain a temperature that approached the value measured by the thermocouple. Then, the emissivity that corresponds to this temperature was deemed as the accurate emissivity, which will be used in the following tests. Images were extracted from each video and processed using a program written in MATLAB code to obtain the flame height. As shown in Figure 1, K-type thermocouple 1 and thermocouple 2 were respectively arranged at the point where the second and third sample plates coincide to measure the surface temperature of the specimen plate. The thermocouples were produced by Hequan Instrument Technology Co. Ltd. (Suzhou, China). The response time of the thermocouple is 1 s, the measurement range is 0–1000 °C, and the accuracy is ±2.2 °C.

At the beginning of the experiment, the end of the PMMA was ignited with an alcohol lamp, then removed the lamp after ignition. In order to reduce the experimental error, experiments were repeated at least 3 times for each experimental condition.

## 3. Results and Discussion

### 3.1. Flame Shape and Structure

**Figure 2:**
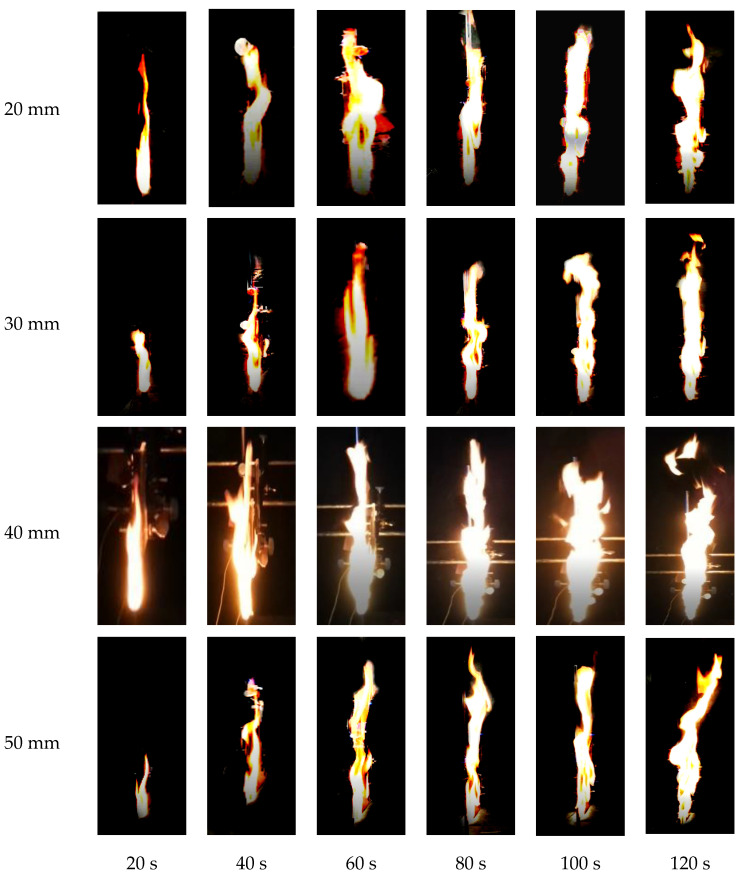
Flame shape and structure under different time and overlap lengths.

Figure 2 shows flame shapes of PMMA with different overlap lengths, i.e., 20 mm, 30 mm, 40 mm and 50 mm. It can be seen from this figure that during the process of flame spread over vertical discrete PMMA, the flame has experienced an incremental process from the ignited sample plates mainly relying on discontinuous flame spread in heat radiation to stable and continuous combustion. In the initial stage of flame spread, the flame adheres closely to the PMMA plate and there is no significant flame fluctuation, which is consistent with Jiang et al.’s results concerning upward flame spread over continuous PMMA plate [17]. As the burning time extends, the shape of the flame changes from an initial rectangle to a jagged shape, a significant periodic flame pulsation can be seen and a great increase in the intensity of flame fluctuation can also be observed. Similar phenomena were observed in the study of Meng et al. [18], who investigated upward flame spread characteristics of discrete thermal insulation material under the influence of porosity. When two or three PMMA plates are in combustion simultaneously, the phenomenon of flame fusion is observed. This phenomenon was also found in the study of Zhang et al. [19], who investigated the flame characteritics of double pool fire.

As the overlap length increases, the PMMA combustion becomes more intense. At the same time, it can be seen that the flame height and flame area become larger correspondingly. The reason is that a larger overlap length of the PMMA plate leads to higher pyrolysis rate, and thus there is sufficient combustible gas released, causing the fully intense burning. Moreover, a decrease in the intensity of combustion at the same time period is observed when the overlap length is larger than 40 mm, compared with that under other experimental conditions. Firstly, this might be attributed to the fact that the overlap length is so large that it can be ignited by the heat radiation of inner flame of the front plate under the lower part of the spreading plate. Secondly, when the overlap length is larger than 40 mm, the end of the spreading plate will be directly ignited by the outer flame of the front plate, leading to its pyrolysis and flame spread.

When the PMMA sample burns to the end, entrainment phenomenon will appear in the top flame. Especially when the overlap length is larger, the phenomenon becomes more significant. Then part of the fire plume bends because of the air entrainment, which is also one of the factors affecting the flame height.

### 3.2. Flame Height

**Figure 3:**
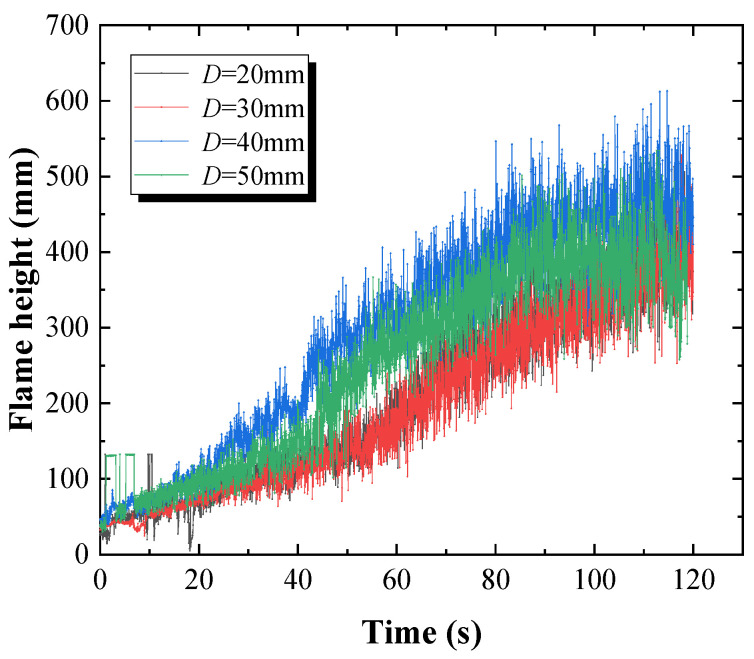
PMMA flame height versus time under different overlap lengths.

The pyrolysis height of vertical PMMA plate array with different overlap lengths are shown in Figure 3. The flame height is mainly determined by the rising height of pyrolysis gas under the effect of buoyancy and the ignition point when it mixes with the air [20,21]. Figure 3 shows that the flame height was small, and the fluctuation of the flame was not obvious in the initial stage. It increased gradually, while sudden changes happened under some experimental conditions. As the PMMA flame continued to spread, the flame height fluctuated more and more significantly. Especially in the stage when the flame of one PMMA plate spreads to the adjacent plate, i.e., the discrete flame spread stage, the flame height changed obviously. The overall increasing of flame height was observed. This is mainly because in the discrete flame spread stage, the adjacent PMMA plate is ignited by heat radiation, and the flame is not in the stable combustion stage, intensifying the fluctuation of the flame height.

In addition, the videos recording flame spread over discrete PMMA were processed using a MATLAB program proposed by us to obtain the real-time flame height. Further, the average flame height of vertical PMMA plate array with different overlap lengths was calculated and presented in Table 5 [22]. Moreover, the standard deviation of flame height is calculated from Figure 3, and the results are also shown in Table 5. The standard deviation could reflect the dispersion degree of the data, and then reflect the fluctuation of the flame height.

It can be deduced from Table 5 that with the increase of overlap length, the average flame height increases gradually first, and then decreases obviously after 40 mm. This is different from the conclusions of Zhao et al. [23], who investigated the effects of sample thickness on the flammability of continuous PMMA plate. They found that the average flame height increased with an increase in sample thickness. Miller et al. [14] studied the flame spread behavior over discrete PMMA plates, which were separated with several noncombustible plates. They defined a parameter named “fuel coverage” as the ratio of noncombustible plate length to the total length of discrete PMMA plates. They also found that the average flame height increased first and then decreased with the increase of the fuel coverage. It demonstrates that when the overlap length is large, the flame height is significantly affected by the overlap length. The difference in the average flame height between 20 mm and 30 mm overlap length is 10.79 mm. This means that when the overlap length is smaller than 30 mm, the influence of the overlap length on the flame height is not significant. However, the difference is 79.31 mm between 30 mm and 40 mm overlap length, and the difference is 39.02 mm between 40 mm and 50 mm overlap length. This indicates that when the overlap length is larger than 30 mm, the effects of the overlap length on the flame height is more significant. In addition, as the overlap length increases, the standard deviation of flame height approximately increases first and then decreases after 40 mm, indicating the similar changing trend of flame fluctuation. This demonstrates that the flame fluctuation is more significant for *D* = 40–50 mm, compared to that when *D* = 20–40 mm.

The non-linear change of the average flame height with the overlap length can be attributed to the coupling of stack effect and heat release rate. Firstly, a partially enclosed vertical channel is formed when two adjacent plates overlap with a certain spacing, and thus the stack effect occurs, which could promote the air flow in the channel. According to An et al.’s work [24], the flow velocity of the induced airflow in the vertical channel is:

(1) $$u=\sqrt{\frac{{2(T}_{v}-T_{\infty })gH}{T_{\infty }{(c}_{in}{+c}_{out}+F\frac{H}{D_{h}})}}=\sqrt{\frac{{2(T}_{v}-T_{\infty })g}{T_{\infty }}}\times \sqrt{\frac{1}{\left(\frac{c_{in}{+c}_{out}}{H}+F\frac{\left(W+L\right)}{2WL}\right)}}$$

where *H* is the height of the vertical channel, $D_{h}$ is the hydraulic diameter of the vertical channel, and $D_{h}=\frac{2WL}{W+L}$. In this paper, the height of the channel is equal to the overlap length, i.e., *H* = *D*. As the overlap length rises, the flow velocity of the induced airflow increases according to Equation (1), further leading to the increase of the average flame height. However, when the overlap length increases to a certain value, the heat release rate may become the dominant factor determining the average flame height. Zukoski et al. [25] proposed the relation between the flame height and heat release rate, as shown in Equation (2).

(2) $$\frac{h_{total}}{h}\approx 16.8\frac{Q_{D}^{*2/5}}{{\left[∆h_{c}/{cT}_{\infty }\right]}^{\frac{3}{5}}{\left({1-X}_{r}\right)}^{\frac{1}{5}}}-1.67$$

where $h_{total}$ is the total flame height, and *h* is sample characteristic length. In this paper, *h* = 100 mm. $Q_{D}^{*}$ is the dimensionless heat release rate, $∆h_{c}$ denotes heat of combustion, and $c_{p}$ is specific heat. $T_{\infty }$ is ambient temperature, and $X_{r}$ is radiation fraction. When the overlap length continues to increase after 40 mm, mutual limiting effects of two adjacent PMMA plates on air entrainment cause inadequate oxygen supply, which leads to inadequate combustion and a lower heat release rate. According to Equation (2), the lower heat release rate corresponds to the lower flame height. Therefore, the average flame height decreases with the increase of overlap length after 40 mm.

### 3.3. Flame Spread Rate

**Figure 4:**
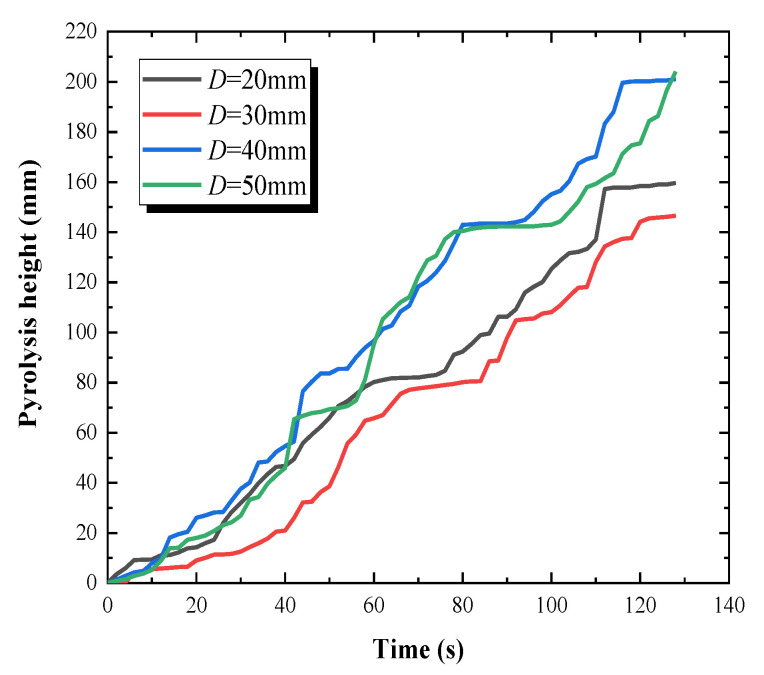
Pyrolysis height versus time.

The pyrolysis height of vertical PMMA plate array with different overlap lengths are shown in Figure 4. The mothed employed to obtain Figure 4 is explained as follows. Firstly, an infrared video was obtained using an infrared camera. Secondly, the infrared pictures were selected 25 frames per second from the infrared video. Thirdly, the pyrolysis height of each infrared picture would be obtained through the CAD ratio method. Finally, the pyrolysis heights under different time were obtained and are depicted in Figure 4.

Conducting linear curve fitting of Figure 4, it can be obtained that the flame spread rates (i.e., the slope of the fitting line) of PMMA plate array under 20 mm, 30 mm, 40 mm and 50 mm overlap lengths are 1.32 mm/s, 1.34 mm/s, 1.67 mm/s, 1.55 mm/s, respectively. With the increase of overlap length, the discrete flame spread rate increases first and then decreases. The flame spread rate for 40 mm overlap length is much larger than that of 20 mm and 30 mm, while there is little change in flame spread rate as the overlap length rises from 20 mm to 30 mm. This phenomenon demonstrates that the flame spread rate will be influenced significantly with the overlap length when it increases to a certain value [26]. This influence can be maximized as the overlap length is 40 mm, while there is a significant reduction in the impact between the overlap length from 20 mm to 30 mm.

When the overlap length is 40 mm, the flame spread rate reaches the maximum. From the flame shape in Figure 2, it is found that when the overlap length reaches 40 mm, the flame of the front plate begins to touch the end of the spreading plate. The temperature of the outer flame is about 500 °C, while the pyrolysis temperature of PMMA plate in this experiment is 350 °C. Therefore, the PMMA plate end will be ignited by direct heating on the basics of the heat radiation, leading to a sharp rise in the flame spread rate.

Further, the reason for the changing trend of flame spread rate is explained. The discrete flame spread rate is closely corelated with the ignition time ($t_{ig}$) of the adjacent PMMA plate. The ignition times under different experimental conditions obtained from infrared video are listed in Table 6. As the overlap length increases, the ignition time first decreases and then increases. When *D* = 40 mm, the ignition time is the shortest. This demonstrated that the discrete flame spread rate is negatively corelated with the ignition time.

The prediction model of ignition time of PMMA plate exposed to constant uniform heat flux was proposed by previous researcher [27]:

(3) $$t_{ig}=\frac{\pi }{4}\kappa \rho c\frac{{{(T}_{ig}-T_{\infty })}^{2}}{{\left(q^{\prime \prime }\right)}^{2}}$$

where κ is heat conduction coefficient of air. ρ and c denote the density and specific heat capacity of PMMA, respectively. $T_{ig}$ is the ignition temperature of PMMA, $T_{\infty }$ is the ambient temperature, and $q^{\prime \prime }$ is the external heat flux.

From Equation (3), the ignition time is negatively corelated with the heat flux. Therefore, it is deduced that the flame spread rate of discrete PMMA plate is mainly determined by the heat flux transferred to its surface. The heat flux under different experimental conditions will be calculated in Section 3.4.

**Figure 5:**
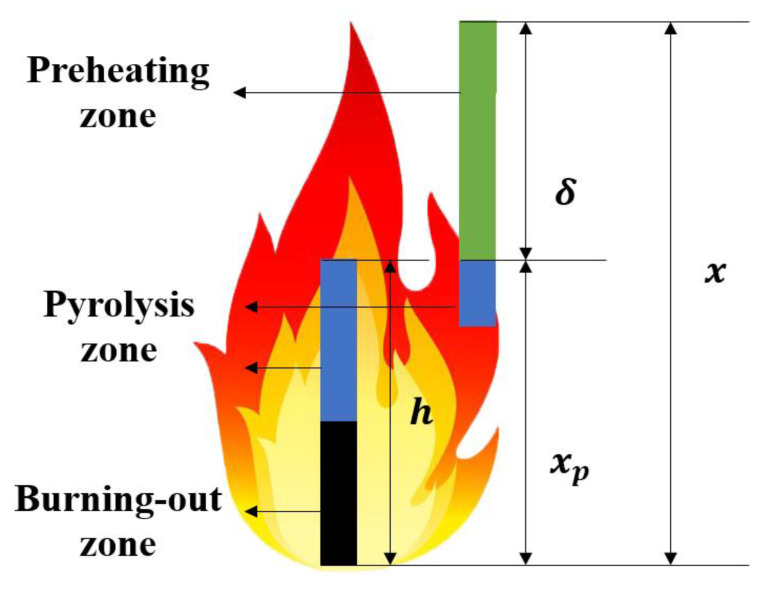
The physical model of vertical flame spread over discrete PMMA array.

Further, the model establishing for predicting flame spread rate of discrete PMMA array is presented as follows. Based on the phenomena observed in the experiment and the continuous fire spread model established by previous researchers, the physical model of vertical flame spread over discrete PMMA array is established, as shown in Figure 5.

For both discrete flame spread and continuous flame spread, the length of the preheating zone is the flame height subtracting the pyrolysis height, i.e., Equation (4). The relation between the flame height and pyrolysis height is Equation (5) [28].

(4) $$\delta =x-x_{p}$$

(5) $${x=kx}_{p}^{n}$$

where δ is the preheating zone length, x is the flame height, and $x_{p}$ is the pyrolysis front height, respectively. The values of *k* and *n* can be found in Hasemi ’s work [28].

Based on the flame spread rate model of a continuous solid proposed by Quintiere [29], this paper establishes the mathematical model for flame spread rate of discrete PMMA array, assuming that the flame heat flux ($q^{\prime \prime }$) received by PMMA surface within the preheating zone is constant. Quintiere proposed the following equation to calculate the flame spread rate of continuous thermal-thick solid.

(6) $$V_{f}^{*}=4\frac{\delta (q^{\prime \prime }{)}^{2}}{{\pi (\kappa \rho c)(T}_{ig}-T_{\infty }{)}^{2}}$$

Substituting Equation (3) into Equation (6), Equation (7) is obtained.

(7) $$V_{f}^{*}=\frac{\delta }{t_{ig}}$$

Miller et al. investigated the correlation between discrete and continuous flame spread rate [14]. In their work, several noncombustible plates were inserted into the PMMA plate, and they defined a parameter named “fuel coverage” as the ratio of noncombustible plate length to the total length of discrete PMMA plates. Further, the relation between discrete flame spread rate $V_{f}$ and $V_{f}^{*}$ was proposed by Miller et al., as shown in Equation (8).

(8) $$V_{f}=\frac{V_{f}^{*}}{f}$$

where f is the fuel coverage. In this work, noncombustible plates were not inserted into the PMMA plate. On the contrary, the discrete PMMA plates overlapped with a certain length (*D*). Therefore, f in this work is defined as follows.

(9) $$f=1+\frac{D}{2h-D}$$

where *h* is the PMMA plate length. Substituting Equations (4), (5), (7), and (9) into Equation (8), Equation (10) is obtained.

(10) $$V_{f}=\frac{x-\sqrt[{n}]{\frac{x}{k}}}{(1+\frac{D}{2h-D}{)t}_{ig}}$$

**Figure 6:**
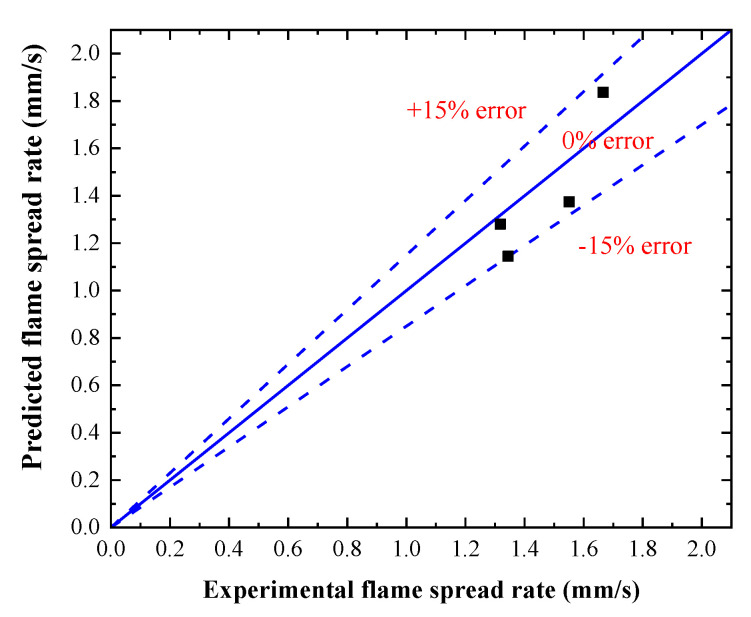
Comparison between predicted and experimental flame spread rate.

Employing Equation (10), the average value of *x* in Table 5, and the data of $t_{ig}$ in Table 6, the predicted flame spread rate of discrete PMMA array can be calculated. Both the calculated and experimental flame spread rate are shown in Figure 6. From Figure 6, it is found that the changing trend of predicted flame spread rate with the overlap length is consistent with the experimental one. The prediction error is smaller than %, within the acceptable error range of engineering.

### 3.4. Temperature Field

**Figure 7:**
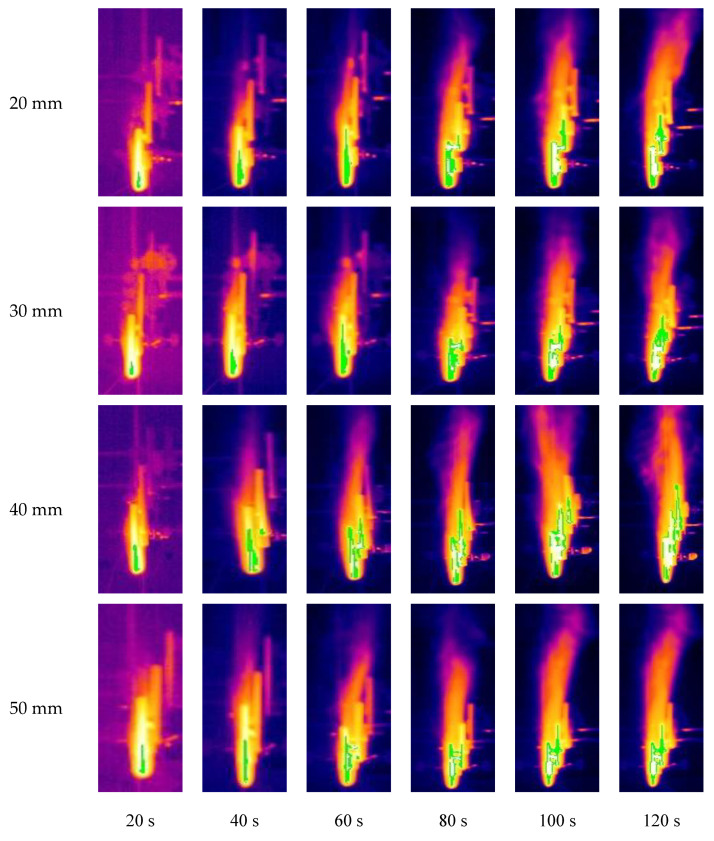
Front view of combustion temperature field.

Figure 7 shows a front view of the temperature field of vertical PMMA plate array with different overlap lengths during combustion. The green part in Figure 7 is the pyrolysis zone of PMMA plate, with a temperature of 350 °C. When the PMMA is ignited, the pyrolysis zone gradually expands and spreads to adjacent PMMA plate [30]. At 120 s, for the overlap lengths of 40 mm and 50 mm, the phenomenon that the pyrolysis area spreads to the third PMMA plate was observed. Among them, the pyrolysis area accounted for about half of the third plate area for 50 mm overlap length, while the third plate in the 40 mm overlap length have completely become the pyrolysis area. For the overlap length of 40 mm, the width of the pyrolysis area of the first two plates was obviously larger than other experimental conditions, and the PMMA plate was sufficiently pyrolyzed. At the 120 s, for the overlap lengths of 20 mm and 30 mm, there is no pyrolysis area spreading to the third plate. It indicates that larger overlap length corresponds to higher flame spread rate, which leads to the increase of the flame height and flame area. At 100 s, only the pyrolysis area under the 40 mm overlap length spreads to the third plate while the pyrolysis area of other three experimental conditions remains in the second plate. it can explain that the discrete flame spread rate decreases as the overlap length rises when it is relatively large [31].

After the PMMA plate is ignited, the pyrolysis area gradually increases, and the flame height and flame area also gradually rise. In addition, the flame of PMMA plate array with a larger overlap length can spread to adjacent plates without complete pyrolysis [32]. For example, for 40 mm overlap length at 40 s, the green area of the first plate only accounts for half of the overall plate area, while in the second plate the green area has already spread to the top of the plate. On the contrary, for the experimental condition with a smaller overlap length, the discrete flame spread is very difficult to keep. For 20 mm and 30 mm overlap length at 120 s, the first two plates were nearly completely pyrolyzed, while the pyrolysis was not observed in the third plate.

**Figure 8:**
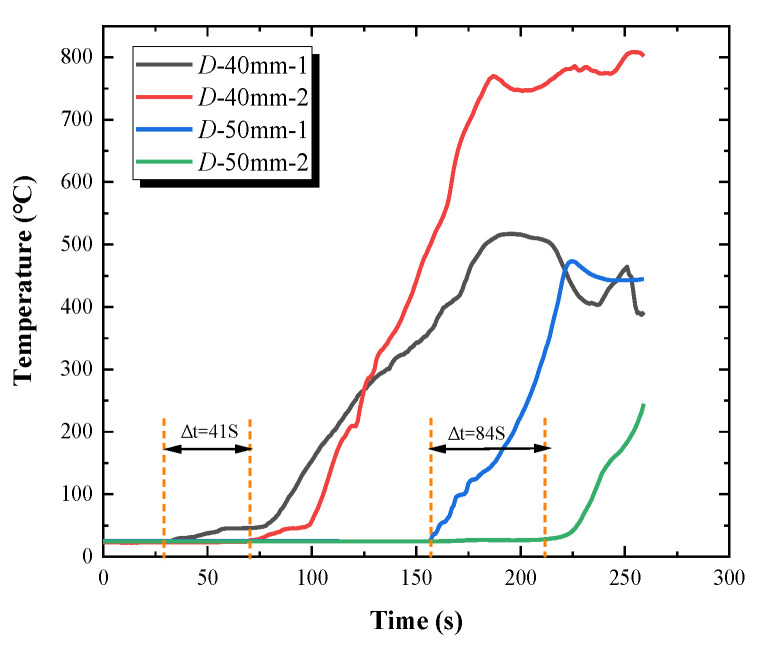
Surface temperature curves of PMMA plate array with 40 mm and 50 mm overlap length.

As shown in Figure 8, for the 40 mm overlap length, the difference in the time for temperature rising between thermocouple 2 and thermocouple 1 was smaller among the four experimental conditions. The temperature change indicates the flame spread rate, which shows the maximum value of the discrete flame spread rate is observed when the overlap length is 40 mm.

**Figure 9:**
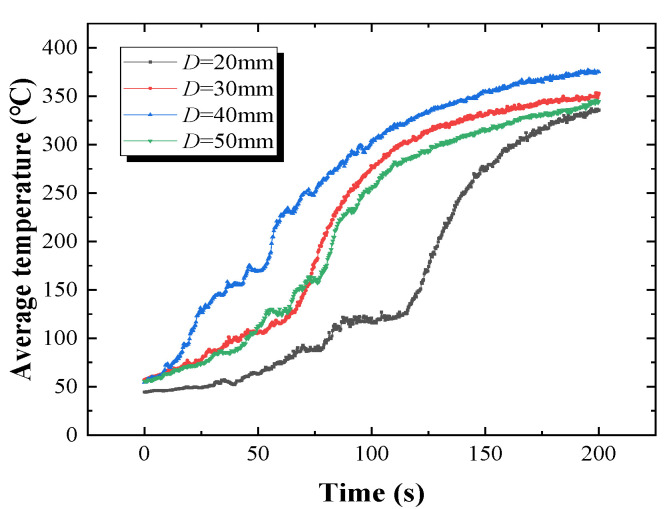
Average temperature of flame zone under different overlap lengths.

The temperature field can be further processed with the software of the infrared camera. The flame area was selected, and the average temperature of this area could be obtained using the IR software. The results are shown in Figure 9, which demonstrates that the average temperature increases first and then decreases as the overlap length rises, reaching the maximum value when *D* = 40 mm.

Further, the average temperature can be used to calculate the radiative heat flux from the flame to the preheating zone of PMMA. According to the Stefan–Boltzmann’s law, the radiant heat flow received by the preheating zone from the opposite flame can be expressed as:

(11) $$q_{rad}=F_{ij}{\sigma \varepsilon }_{f}T_{f}^{4}$$

where $F_{ij}$ is the view factor, σ is the Boltzmann’s constant, $\varepsilon_{f}$ is the emissivity of PMMA. σ = 5.67 × $10^{-8}\;W^{2}\;m^{-4}{\;K}^{4}$ and $\varepsilon_{f}$ = 0.92 [33]. In this work, the average value of the average temperature shown in Figure 9 is deemed as $T_{f}$.

The view factor can be obtained using the following integration:

(12) $$F_{ij}=\frac{1}{A_{i}}\int_{A_{i}}\int_{A_{j}}\frac{{sin\theta }_{i}{cos\theta }_{j}}{{\pi R}^{2}}{dA}_{i}{dA}_{j}$$

where $\theta_{i}$ and $\theta_{j}$ denote the angle between the radiation ray and the straight line perpendicular to the elements ${dA}_{i}$ and ${dA}_{j}$, *R* is the distance between the elements ${dA}_{i}$ and ${dA}_{j}$. For two parallel plates with a certain spacing (*L*) and overlap length (*D*), Equation (12) could be transformed to the following equation:

(13) $$F_{ij}=\frac{{\left[{\left(h+D\right)}^{2}{+4L}^{2}\right]}^{1/2}-{\left[{\left(h-D\right)}^{2}{+4L}^{2}\right]}^{1/2}}{2D}$$

where L = 8 mm and *h* = 100 mm. Substituting Equation (13) into Equation (11), the radiative heat flux from the flame to the preheating zone of PMMA can be obtained and shown in Table 7. As discussed in Section 3.3, the flame spread rate of discrete PMMA plate is mainly determined by the heat flux transferred to its surface, including radiative and convective heat flux. Zhao et al. [34] indicated that for upward flame spread over vertical PMMA plate, the convective heat transfer plays an important role when the spacing is small, while the radiant heat transfer is dominant when the spacing is larger. The plate spacing in this work is 8 mm, and thus the flame spread rate is mainly determined by radiant heat transfer. From Table 7, the radiative heat flux increases first and then decrease as the overlap length rises, reaching the maximum value when *D* = 40 mm. This could explain the changing trend of the flame spread rate in Section 3.3.

## 4. Conclusions

The effects of overlap length on the flammability and flame spread of vertical PMMA plate array were investigated in this work. Flame shape, flame height, flame spread rate, and temperature distribution were measured and discussed. The influence mechanism of overlap length is revealed. The following conclusions are drawn:

(1) At the initial stage of flame spread over vertical PMMA plate array, the fluctuation of flame is not obvious while it is intensified at the later stage. The flame height and flame size generally increase as the flame spreads. As the overlap length increases, the average flame height increases first and then decreases, and reaches the maximum when *D* = 40 mm. The non-linear change of the average flame height can be attributed to the coupling of stack effect and heat release rate.
(2) The discrete flame spread speed also rises first and then drops with the increase of overlap length, which mainly due to the heat transfer from the PMMA flame to the adjacent plate. As the overlap length rises, the heat flux rises first and then drops, reaching the maximum when the overlap length is 40 mm. A model for predicting the flame spread rate of a discrete PMMA array is established. The predicted changing trend of the flame spread rate is consistent with the experimental one. The prediction error is smaller than 15%.
(3) For larger overlap length, the pyrolysis area of combusting PMMA plate is smaller when the next PMMA plate is ignited. As the overlap length increases, the time when PMMA surface temperature begins rising is advanced, and the growth rate of the surface temperature is larger. The average temperature of flame zone rises first and then drops with the increase of overlap length. Radiative heat flux to the preheating zone is calculated, and its changing trend with the overlap length is the same as that of the average temperature.

Results obtained in this work are beneficial to fire hazard evaluation and fire safety design of buildings or industrial sites employing PMMA. The fire hazard evaluation will help safety management and conducting fire insurance of factories and buildings related to PMMA material. Moreover, results of this work are helpful to determine the optimal fire protection distance between PMMA plates.

## Figures and Tables

**Table 1:** Experimental environmental conditions.

Experimental Site	Elevation (m)	Atmospheric Pressure (kPa)	Absolute Oxygen Concentration (kg/m^3^)	Ambient Temperature (°C)	Ambient Humidity (%)
Xuzhou city	42	101.2	0.267	15–23	30–45

**Table 2:** Parameters of PMMA physical characteristics.

	Property	Quantity
*B*	mass transfer number	1.32
k	thermal conductivity of gas phase	0.05 ${\mathrm{Wm}}^{-1}K^{-1}$
ρ	density	1190 ${\mathrm{Kg}\;m}^{-3}$
*c*	specific heat capacity	1400 ${J\;\mathrm{Kg}}^{-1}K^{-1}$
$T_{g}$	vitrification temperature	104 °C
$T_{m}$	melting temperature	270 °C
$T_{p}$	pyrolysis temperature	350 °C
$T_{f}$	flame temperature	800 °C
*Δ* $h_{c}$	heat of combustion	24.9 KJ/g
$P_{r}$	Prandtl number	0.703
σ	Stefan-Boltzmann constant	5.67 × $10^{-8}{\;W}^{2}{\;m}^{-4}{\;K}^{4}$
α	thermal diffusivity	${168\times 10}^{-6}{\;m}^{2}{\;s}^{-1}$

**Table 3:** Experimental condition.

Experimental Condition	1	2	3	4
*D* (mm)	20	30	40	50

**Table 4:** Performance parameters of cameras used in this work.

Camera	Performance Parameter	Parameter Value
	type	(SONY) HDR-CX450
	resolution ratio	1920 × 1080
Digital camera	frame rate	25 fps
	focus method	auto/manual
	maximum aperture	F1.8–F4.0
	type	MAG30HT
	resolution ratio	384 × 288
Infrared camera	frame rate	100 fps
	wavelength range	7.5~14 μm
	temperature range	−20~1200 °C
	accuracy	2%

**Table 5:** Average value and standard deviation of flame height of vertical PMMA plate array with different overlap length.

Overlap Length (mm)	20	30	40	50
Average Flame Height (mm)	196.46	207.25	286.56	247.55
Standard Deviation	123.14	117.94	147.02	126.16

**Table 6:** Ignition times of the second and third PMMA plate under different experimental conditions.

Overlap Length (mm)	20	30	40	50
$t_{ig}$ (second plate) (s)	60	52	24	34
$t_{ig}$ (third plate) (s)	116	112	69	93

**Table 7:** View factor and radiative heat flux from the flame to the preheating zone.

**Overlap Length (mm)**	20	30	40	50
**View Factor**	0.986941	0.986260	0.985182	0.983533
**Radiant Heat Flux (W/m^2^)**	4176.9	4348.7	5563.2	3025.8
